# Older patients with ANCA-associated vasculitis and dialysis dependent renal failure: a retrospective study

**DOI:** 10.1186/s12882-015-0082-9

**Published:** 2015-06-25

**Authors:** Rebecca L. Manno, Philip Seo, Duvuru Geetha

**Affiliations:** Division of Rheumatology, Johns Hopkins Vasculitis Center, Johns Hopkins University School of Medicine, 5501 Hopkins Bayview Circle, Room 1B.13, 21224 Baltimore, MD USA; Division of Nephrology, Johns Hopkins Vasculitis Center, Johns Hopkins University School of Medicine, Baltimore, MD USA

**Keywords:** ANCA, Elderly, Vasculitis, Hemodialysis

## Abstract

**Background:**

ANCA-associated vasculitis (AAV) with renal involvement is not uncommon in older individuals. Unfortunately, this can be catastrophic requiring hemodialysis (HD) and may lead to end stage renal disease (ESRD). However, more than 50 % of patients with AAV who require HD initially have renal recovery and discontinue HD. The aim of this study was to describe a retrospective cohort of older patients with AAV and severe renal involvement which required hemodialysis.

**Methods:**

Between 1995 and 2013 a total of 30 patients with histologic evidence of pauci-immune glomerulonephritis who required HD were evaluated at a single university center. The association of demographic and clinical parameters with age was assessed. Older age of disease onset was defined as age ≥60 years. The risk of developing ESRD at 3 months was examined using univariate logistic regression analysis.

**Results:**

Among 30 patients with AAV who required HD, the mean age of disease onset was 59 ± 17 years (range 22-88 years). Twelve patients were in the older age group, and 18 were in the younger group. Three months after diagnosis, 43 % of the cohort had ESRD with a statistically similar proportion of older (n = 9, 50 %) versus younger (n = 4, 33 %) patients (p = 0.367). Most patients (93 %) received immunosuppressive therapy. There was not a statistically significant association between age and ESRD.

**Conclusions:**

These data suggest that age alone does not predict renal recovery among individuals on HD due to AAV. Renal recovery is a realistic expectation and outcome, if patients are treated, even among older patients with AAV who require HD initially.

## Background

The population of the United States is aging. It is anticipated that the population of Americans aged 65 years or older will double during the next 25 years to about 72 million [1]. Although ANCA-associated vasculitis (AAV) is rare in the general population, epidemiologic data demonstrate that it has a predilection for older individuals with some studies citing a peak incidence in the 65–74 year age group [2, 3]. Therefore, it is expected that this demographic will be increasing in the very near future.

Renal involvement in AAV is common and can be severe requiring renal replacement therapy [4]. This has important implications as severe renal dysfunction in AAV is associated with poor survival and increased risk for progression to end stage renal disease (ESRD) [5, 6]. The combination of older age and severe renal involvement from AAV portends a particularly grim outcome [7–10].

It is well established that without treatment AAV leads to considerable morbidity and mortality. Conversely, if treatment is appropriately initiated, less than 40 % of patients who present with severe kidney failure from AAV which requires hemodialysis will remain dialysis dependent [11]. Recent data have demonstrated that although low baseline glomerular filtration rate (GFR) and high burden of renal scar on biopsy are associated with decreased treatment response rates, there is no identifiable GFR cutoff or clear pathologic finding where treatment would be considered futile [11–13]. However, the potential toxicities of immunosuppressive therapy, such as infection, cytopenias and drug interactions can be daunting when considering treatment for an older patient with severe AAV renal disease on hemodialysis. This vulnerable population may be particularly susceptible to the adverse effects of therapy.

Histopathologic classification of glomerulonephritis in AAV has emerged as a potential predictor of long-term renal outcomes [13]. However, recent data has questioned if histologic class alone provides additional clinically useful information beyond established predictive parameters, such as age, baseline GFR and degree of tubular atrophy [14].

The aim of this study was to describe a retrospective cohort of older patients with AAV and severe renal involvement which required hemodialysis. A younger cohort of patients with AAV and severe renal involvement was included for comparison.

## Methods

### Study population

Potential patients for this study were identified from a pathology database (1995–2013) using the term “pauci-immune glomerulonephritis.” To be included in the study, individuals had to have a pathologic (kidney biopsy) and clinical diagnosis of AAV.

AAV was defined as granulomatosis with polyangiitis (GPA) or microscopic polyangiitis (MPA). Patients had to require hemodialysis at the time of kidney biopsy and were followed for at least twelve months after biopsy. This study protocol was approved by the Johns Hopkins Office of Human Subjects Research and Institutional Review Board. Since this was a retrospective observational study for the generation of a de-identified data set, written informed consent of each participant was not required.

### Acquisition of clinical and laboratory data

Patient demographics, clinical features at the time of diagnosis (including age), treatment regimens, hemodialysis duration, laboratory data and outcomes were abstracted retrospectively from the medical record.

### Definitions

Patients were classified as having GPA or MPA based on the Chapel Hill Consensus Conference Criteria [15]. Age at the time of diagnosis was determined from the medical record, and late-age onset AAV was defined as the onset of disease at age 60 years or older. The outcome of ESRD was defined as the need for continued dialysis for more than 3 months. Late ESRD was defined as the resumption of renal replacement therapy after *discontinuation* of hemodialysis within the first three months. Renal recovery was defined as the discontinuation of hemodialysis for any period of time as documented by the treating physician in the medical record. Renal histopathology was grouped into four classes based on criteria used by the *International Working Group of Renal Pathologists* [13]: Focal (≥50 % normal glomeruli), Crescentic (≥50 % glomeruli with cellular crescents), Mixed (<50 % normal, <50 % crescentic, <50 % globally sclerotic glomeruli), and Sclerotic (≥50 % globally sclerotic glomeruli).

### Statistical analysis

Continuous, normally distributed variables were summarized as mean value ± standard deviation. Discrete variables were summarized as proportions. Differences in means between the older and younger cohorts were evaluated using non-parametric tests for continuous variables (Wilcoxon-Mann-Whitney). Differences in proportions between cohorts were evaluated with Chi-square test for discrete variables. Logistic regression was used to calculate odds ratios and determine the risk of ESRD as a function of age at diagnosis. Age was modeled as a continuous variable, dichotomous variable (cut-off ≥60 years) and by tertiles (<60 years, 60–69 years, ≥70 years). Statistical analyses were performed using Stata IC 10.0. *P*-values are two sided with α = 0.05.

## Results

The baseline characteristics of the 30 patients who met inclusion criteria for this study are presented in Table 1. The mean age of AAV onset among the cohort was 59 ± 17 years (range 22 to 88 years). Eighteen patients (60 %) had late-age onset AAV, defined as disease onset at age 60 years or greater. Of those with late-age onset AAV, 10 (56 %) had disease onset between ages 60 and 70 years, 6 (33 %) had disease onset between 71 and 79 years, and 2 (11 %) had onset after age 80. The majority of the cohort had a new diagnosis of AAV (n = 26, 87 %). Among those with relapsing disease (n = 4), 3 patients were in the late-age onset group.

**Table 1:** Baseline characteristics of patients with ANCA-associated vasculitis who required hemodialysis at the time of kidney biopsy stratified by age of vasculitis diagnosis

	Age of onset < 60 years (n = 12)	Age of onset ≥ 60 years (n = 18)	*p*-value
Age at diagnosis, years (mean ± SD^a^)	41 ± 11	71 ± 7	<0.001
Female	8 (67 %)	7 (39 %)	0.136
African-American	4 (30 %)	2 (11 %)	0.136
Diagnosis			
Granulomatosis with polyangiitis	8 (67 %)	10 (56 %)	0.543
Microscopic polyangiitis	4 (33 %)	8 (44 %)	
PR3-ANCA positive^b^	6 (67 %)	8 (50 %)	0.420
MPO-ANCA positive^b^	3 (33 %)	8 (50 %)	
Peak serum creatinine, mg/dL (mean ± SD^a^)	5.8 ± 2.5	8.2 ± 4.4	0.150
Normal glomeruli, % (mean ± SD^a^)	21.5 ± 18.1	27.4 ± 23.8	0.432
Renal histopathologic class			
Crescentic	4 (34 %)	4 (22 %)	
Focal	1 (8 %)	3 (17 %)	
Mixed	6 (50 %)	9 (50 %)	
Sclerotic	1 (8 %)	2 (11 %)	

^a^ *SD* standard deviation

^b^Data analyzed from 25 individuals who were ANCA positive

All of the patients had severe renal involvement at presentation requiring hemodialysis. The mean peak creatinine was higher among those with late-age onset AAV (8.2 ± 4.4, *older* vs 5.8 ± 2.5 mg/dL, *younger*; *p* = 0.150), although this did not reach statistical significance. The histopathologic classes were similarly distributed between the older and younger vasculitis patients, with half of the patients in each age group with Mixed pathology. Both older and younger patients had similar percentage of normal glomeruli on biopsy.

All 30 patients in this cohort had some extra-renal manifestation of vasculitis. An equal proportion of patients in the older (72 %) vs the younger (75 %) age group had lung involvement attributed to their vasculitis (*p* = 0.866), although few had respiratory failure (17 % older vs 22 % younger, *p* = 0.709). Fewer older patients (28 %) had inflammatory arthritis attributed to vasculitis at presentation compared to younger patients (58 %) and no patients with AAV onset after at 70 years had inflammatory arthritis at the time of presentation.

All 30 patients in the cohort were treated with corticosteroids initially with the majority (87 %) receiving intravenous methylprednisolone. Two patients, both in the late-age onset group, received corticosteroid monotherapy. These two patients had Sclerotic class biopsies without extra-renal disease. In addition, both of these patients were treated at outside facilities and were seen for a second opinion at this tertiary care center.

All (n = 12, 100 %) of the younger patients received cyclophosphamide therapy compared to only 78 % of the older patients. Cyclophosphamide was administered orally among the majority who received it (83 %). Among the older patients, 5 % were treated with rituximab and 5 % were treated with mycophenolate mofetil. All patients over the age of 70 years were treated with cyclophosphamide, except for the oldest patient in the cohort (88 years old) who was the sole recipient of mycophenolate mofetil. A similar proportion of older (28 %) and younger (17 %) patients also received adjuvant plasmapheresis at that time of diagnosis (*p* = 0.481).

Three months after diagnosis, 43 % of the cohort had ESRD with an equal proportion of older (n = 9, 50 %) vs younger (n = 4, 33 %) patients (*p* = 0.367). One patient had late ESRD with onset 4 months after diagnosis. This patient was in the younger age group (age 27 years). Conversely, 67 % of the younger cohort (age onset <60 years) had renal recovery and discontinued hemodialysis within the first 3 months after diagnosis. Similarly, 50 % of those age 60–70 years and 50 % of those age ≥70 also discontinued hemodialysis and had renal recovery. This included the oldest patient in the cohort (age 88 years) who had complete renal recovery and discontinued hemodialysis. The two patients who received corticosteroid monotherapy (both in the late-age onset AAV group) had ESRD and no renal recovery. Among patients treated with cyclophosphamide, 42 % had ESRD. Both patients treated with mycophenolate mofetil and rituximab had renal recovery at three months and were in the late-age onset AAV group.

Within the 12-months of follow up after biopsy, there were two deaths. Both deaths were in the late-age onset group (ages 65 and 69 years). There were no deaths in the first year of follow up in the younger age onset group.

There was no statistically significant association between age and ESRD in univariate logistic regression models. With age modeled as a continuous variable there was not an increased risk of ESRD for a one year increase in age of AAV onset (OR 1.01, *p* = 0.677). When comparing patients with disease onset younger than age 60 to those older (age as a binary variable), there was a trend for an increased risk of ESRD among the older patients (OR 1.55, *p* = 0.370), but this did not reach statistical significance. Similarly, there was a trend for an increased risk of ESRD among the oldest patients with AAV (age ≥ 70 years) compared to the youngest patients (<60 years), OR 1.87 (*p* = 0.459).

## Discussion

There is relatively little information on the clinical features and outcomes of older individuals with AAV on hemodialysis. Our study suggests that age alone does not predict renal recovery among individuals on hemodialysis due to AAV. The majority of patients in our cohort received treatment with aggressive immunosuppressive therapy, which is necessary for renal recovery, regardless of age.

Previous reports which have examined AAV in older populations have not focused specifically on elderly who required hemodialysis at presentation. Bomback, *et al.* described the clinical course of 78 patients over the age of 80 years who had biopsy proven pauci-immune glomerulonephritis but did not necessarily require renal replacement therapy at diagnosis (mean creatinine at biopsy 4.3 ± 2.5 mg/dl) [16]. They found peak serum creatinine and use of immunosuppressive therapy influenced progression to ESRD and that the highest risk of morbidity and mortality for these elderly patients was within the first six months after diagnosis, which suggests that the benefit of immunosuppressive therapy is early in the disease [16]. Similarly, in our cohort, patients who received steroid monotherapy, without an additional immunosuppressive agent, did not have any renal recovery.

De Lind van Wijngaarden, *et al.* prospectively followed 69 patients with AAV who required hemodialysis at the time of diagnosis and evaluated if the hazards of immunosuppressive treatment outweigh expectations of recovery [12]. The mean age of the cohort was 64 years (range 26 to 78). The authors did not focus on age as a predictor in their models, but they found the chance of renal recovery among patients with AAV on hemodialysis was best predicted by type of adjunctive treatment and histopathologic findings. However, even with ominous biopsy features the chance of renal recovery exceeded the chance of therapy-related death if treatment includes plasma exchange [12]. Another study of 155 patients with AAV and severe kidney involvement (GFR < 15 mgl/min per 1.73 m^2^) similarly found that although poor renal function at diagnosis was associated with lower treatment response rate, there was not a “futility” threshold below which the risk of therapy would universally outweigh potential benefits [11].

A study of Chinese patients with AAV on hemodialysis at the time of diagnosis found that 50 % of patients progressed to ESRD [17], which is similar to the findings in our study (46 % patients with ESRD at 6 months). The mean age of this cohort was 57 ± 16 years (range 14 to 83), and they found that older age was associated with increased risk of all-cause and therapy-related mortality but age alone (by one year increments) was not associated with dialysis independence [17].

In our single center experience, which was limited by retrospective design and small sample size, 57 % of patients who required hemodialysis at the time of diagnosis due to AAV had renal recovery. Among the oldest patients in the cohort, 50 % achieved renal recovery, including the oldest patient who was age 88. Similar to the previously published reports, renal recovery in our cohort only occurred in the context of treatment. The two patients who did not receive treatment beyond corticosteroids presumably had additional immunosuppressive therapy held because of their age and kidney biopsy class (Sclerotic). As discussed, age should not be a deciding factor in determining treatment, because even the oldest patient in our cohort was able to achieve renal recovery with appropriate therapy. Regarding the biopsy findings in these two patients (Sclerotic) recent data has demonstrated that even with severe renal scarring there is no “futility” threshold which can be identified for therapy [11]. The unfortunate management of these two patients demonstrates “real world” practices. Hence, the importance of our study, albeit small, to demonstrate success and renal recovery even among our oldest patients when proper treatment was administered.

Less than one-third of our total cohort received adjuvant plasmapheresis, which remains a controversial part of management for AAV with severe renal failure in the absence of alveolar hemorrhage in the United States [18, 19]. As more data has emerged recently about the benefits of this therapy in combination with immunosuppression in severe AAV, plasmapheresis is being used with a greater frequency today than was seen during the study period of our cohort. Future studies investigating the effectiveness of plasmapheresis for AAV should include *older* individuals to assess if there is a particular benefit for this vulnerable population.

Although nearly all patients in this cohort received cyclophosphamide, the optimal induction regimen and dose for older patients with AAV still requires further study. Rituximab was not yet FDA approved during a majority of our study period which is indicated by majority of patients receiving cyclophosphamide. However, cyclophosphamide dosing issues and drug-induced cytopenias are significant concerns for older patients, and the potential benefit of rituximab over cyclophosphamide for the treatment of older individuals with AAV should be explored.

The approach to management of older patients may be ‘less is more’ when there are concerns that the side effects and toxicities of therapy may be worse than the disease itself. The decision to treat an older patient who has already suffered a major complication from AAV, such as renal failure requiring dialysis, is a challenging one. Without treatment, inevitably the patient will be relegated to ongoing renal replacement therapy, and ESRD is inescapable. However, these data show that renal recovery can occur, even among the oldest patients with AAV. Older age and severe renal involvement are predictors of the most ominous outcomes from AAV, and yet renal recovery is still a realistic expectation for this population.

## Conclusions

In summary, these data support that renal recovery is a realistic expectation and outcome among older patients who require HD initially. Age alone is not an accurate predictor of late or definitive ESRD nor is treatment with steroids monotherapy sufficient. We recognize the small sample size of this study and retrospective design as limitations. However, these data provide important insight into some of the complexities physicians are faced with in the management of these very sick patients. Our study has value as a sample of “real world” practices, experiences, and outcomes outside of the clinical trial setting.

## Abbreviations

AAV: ANCA-associated vasculitis
ESRD: End stage renal disease
GFR: Glomerular filtration rate
GPA: Granulomatosis with polyangiitis
MPA: Microscopic polyangiitis
